# Risk factors for wound infection caused by Methicillin Resistant *Staphylococcus aureus* among hospitalized patients: a case control study from a tertiary care hospital in India

**DOI:** 10.4314/ahs.v21i1.37

**Published:** 2021-03

**Authors:** Latha Thimmappa, Anil Bhat, Manjunatha Hande, Chiranjay Mukhopadhyay, Elsa Devi, Baby Nayak, Anice George

**Affiliations:** 1 Manipal College of Nursing Manipal, Manipal Academy of Higher Education Manipal, Karnataka India; 2 Kasturba Medical College, Manipal, Manipal Academy of Higher Education Manipal, Karnataka India; 3 Manipal-McGill Centre for Infectious Diseases, Manipal, Manipal Academy of Higher Education Manipal, Karnataka India

**Keywords:** MRSA, infection, India

## Abstract

**Background:**

Methicillin Resistant *Staphylococcus aureus* (MRSA) causes infection in hospitals and communities. The prevalence and risk factors of MRSA infection is not homogenous across the globe.

**Objective:**

To find the risk factors of MRSA infection among hospitalized patients.

**Methods:**

Cross-sectional case control study was conducted at a tertiary care hospital in India. The risk factors were collected using checklist from 130 MRSA and 130 Methicillin sensitive *staphylococcus aureus* (MSSA) infected patients. The pathogens were isolated from the wound swabs according to Clinical and Laboratory Standards Institute guidelines.

**Results:**

Both the groups were comparable in terms of age, gender, diabetic status, undergoing invasive procedures, urinary catheterization and smoking (p>0.05). Multivariate logistic regression revealed surgical treatment (OR 4.355; CI 1.03, 18.328; p=0.045), prolonged hospitalization (OR 0.307; CI 0.11, 0.832; p=0.020), tracheostomy (OR 5.298, CI 1.16, 24.298; p=0.032), pressure/venous ulcer (OR 7.205; CI 1.75, 29.606; p=0.006) and previous hospitalization (OR 2.883; CI 1.25, 6.631; p=0.013) as significant risk factors for MRSA infection.

**Conclusion:**

Surgical treatment, prolonged and history of hospitalization, having tracheostomy for ventilation and pressure/venous ulcer were the key risk factors. Therefore, special attention has to be given to the preventable risk factors while caring for hospitalized patients to prevent MRSA infection.

## Introduction

Methicillin Resistant *Staphylococcus aureus* (MRSA) is a Gram-positive pathogen, having the ability to cause hospital associated infection and/or community acquired infection. Hospital associated MRSA infection is one of the major problems affecting both patients and care providers^1^. MRSA colonization is predominantly present in the nose and skin of humans^2^. Nasal colonization of *Staphylococcus aureus* and MRSA are the independent predictors of MRSA infection^3^. Colonized bacteria may not cause infection. However, it can enter the body through injured skin or mucus membrane and can cause simple skin infection to life threatening bacteremia. The spectrum includes pneumonia, bacteremia, skin and soft tissue infection, pyomyositis, sepsis, osteomyelitis, necrotizing pneumonia and necrotizing fasciitis^4^. Though MRSA can be isolated from blood, nose, wound, urine, respiratory tract, sputum and other body fluids, the prevalence is high in wounds^5^.

Acquiring MRSA infection is multifactorial, and the risk factors described are prolonged post-operative state, emergency admissions and prior treatment with multiple antibiotics^6^. Other notable treatment related factors are emergency surgery, prolonged or multiple hospital stays, use of invasive devices (catheters, surgical drains, gastric/endotracheal tubes), repeated surgeries, treatment with multiple broad-spectrum antibiotics, inpatient in a neonatal or surgical ICU and poor infection control practices^7^–^12^. The host related factors are age over 65 years, any conditions that suppress immune system function, open wound or injuries, unsanitary or crowded living conditions like dormitories or military barracks, sharing towels or other personal items^7^–^12^. Comorbidities such as diabetes mellitus (66%), hypertension (66%) and sickle cell diseases (33%) are also the threat for acquiring MRSA infection^13^.

MRSA contaminates the hands of healthcare professionals (59.6%)^7^. Even the dress of healthcare professionals can spread MRSA. According to the society for Healthcare Epidemiology of America (SHEA) report (2014), HCPs opine that their attire, including footwear, is important in preventing transmission of infection^14^. Also, MRSA is found on hospital surfaces, disinfectant areas and reusable equipment^15^. Though the cleaning of patient surroundings in ICU has shown a significant reduction in MRSA, after 24 hours of cleaning, the risk of MRSA growth in the patient environment remained high^16^.

Although some similar strains of MRSA are seen in many countries depicting international dissemination, the spread is not homogenous around the globe^17^. Most of the studies have been conducted in developed countries 3,12,18. No published information on risk factors of MRSA infection is traced in India. Therefore, we aimed at identifying the risk factors of MRSA infection in an Indian hospital to institute appropriate preventive measures.

## Methods

### Study design

The study has adopted a cross-sectional case control study design (1:1) with a quantitative approach.

### Study setting

The study was carried out in a tertiary care hospital in South India. The hospital has almost all the super specialties with 2032 beds and provides both in-patient and outpatient healthcare services. It caters to the health needs of a large population. It is a private university hospital meeting the teaching needs of many health science courses such as medical, dental, nursing and other allied health courses. The hospital had more than 80% occupancy during the study period. The hospital is certified by the International Organization for Standardization, (ISO) 14001: 2015 ISO 50001:2011 and accredited by National Accreditation Board for Hospitals & Healthcare Providers (NABH).

### Participants and sample size

Hospitalized patients infected with MRSA were the cases. Patients with Methicillin Sensitive *Staphylococcus aureus* (MSSA) infections were considered as controls.

The sample size for identifying the risk factors of MRSA infection was calculated based on the previous study reports by using the following formula 19.

$$n={\left\{\frac{Z_{1\frac{\alpha }{2}}\sqrt{2pq}+Z_{1-\beta }\sqrt{p_{1}q_{1}+p_{2}q_{2}}}{{\left(P_{1}-P_{2}\right)}^{2}}\right\}}^{2}$$

The proportion at baseline was 0.73 and the expected outcome set was 0.53 (based on the previous hospitalization as the risk factor)^19^. The measured confidence interval was 95% with 80% power, and the calculated sample was 88 in each group. Considering the presence of skin ulcers (baseline 0.33 and expected outcome 0.18)^19^ the calculated sample size was 129. The study included 130 patients with MRSA infection (cases) and 130 patients with MSSA infection (controls). Hospitalized patients who had MRSA grown in their wound culture were considered as cases, whereas hospitalized patients with MSSA grown in the wound swabs were taken as controls. We recruited both male and female adult patients (18 years and above) of general wards, medical and surgical intensive care units. The wards included were medical, surgical, dermatology, orthopedics, cardiology, Ear, nose and throat (ENT) specialties. The patients who were hospitalized for more than two days (>48 hours) as in-patients were included. Patients with immunosuppressive with human immunodeficiency virus, cancer and on immunosuppression therapy were excluded from the study. However, patients with agranulocytosis, leukocytosis and mild autoimmune disorder were not excluded.

### Risk assessment checklist

There was no standardized tool available for identifying the risk factors of MRSA infection. Hence, a checklist of risk factors for MRSA infection was developed after an extensive literature search and discussion with microbiologists and Hospital Infection Control Committee (HICC) members. The checklist had 31 dichotomous items with ‘yes’ or ‘no’ options. The content validity was done by nine experts from different healthcare professionals (members of HICC, microbiologists, physicians, faculty of nursing and a policymaker). Content validity index was 0.94. The reliability of the tool was established by the raterinter-rater method, and the calculated ‘r’ was 0.974.

### Ethical consideration

Ethical permission was obtained from the Institutional Ethics Committee (IEC). The study was registered at ‘clinical trail registry – India’ (CTRI/2018/01/011510). Administrative approval was taken from the Medical Superintendent and Chief Operating Officer of the hospital. Informed written consent from study participants was obtained.

### Data analysis

The data were coded and entered in Statistical Package for Social Sciences (SPSS 16.0) version and the analysis was performed using logistic regression. The demographic characteristics are given in frequency and percentage.

### Data collection procedure

**Figure 1:**
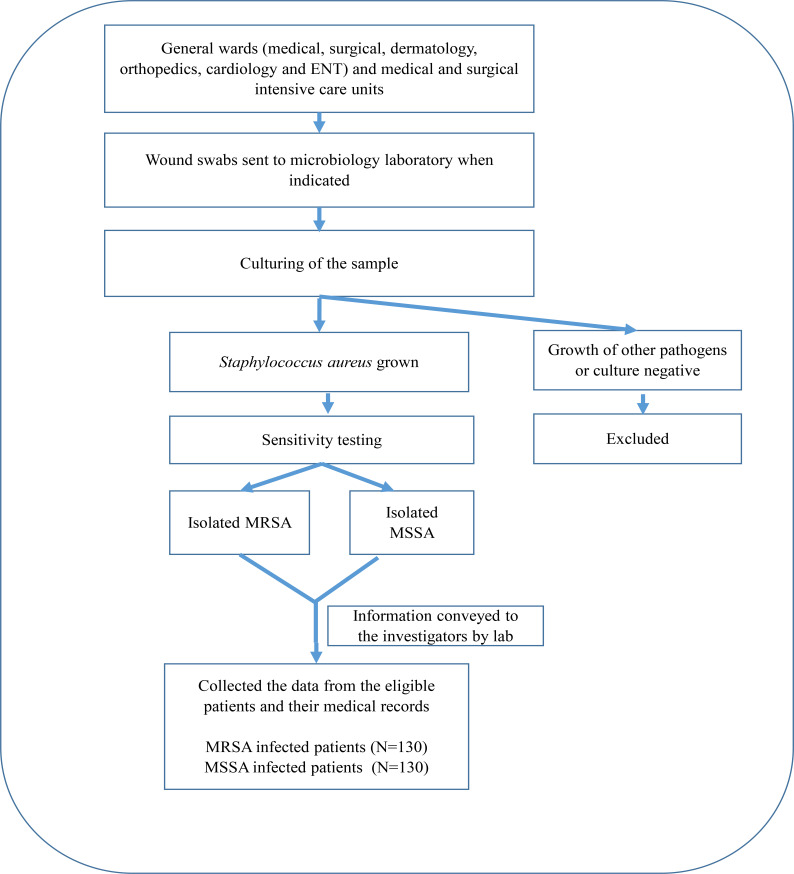
Flow diagram of patient recruitment and data collection

We collected the data from June 2017 to May 2018. The hospitalized patients, whose wound swab grew MRSA or MSSA were approached as presented in the flow diagram (figure 1). After obtaining the consent, investigators collected information from the patients and the medical records using a risk assessment checklist. A total of 260 (130 MRSA infected and 130 MSSA infected) patients were recruited.

## Results

Both MRSA and MSSA infection groups were comparable in terms of age, gender, admission status, immunity, diabetes mellitus, smoking status, having undergone invasive diagnostic procedures, presence of a catheter, feeding tubes and duration of surgery as shown in Table 1. The mean duration of hospital stay was 9.9 days (range: 1–38 days) for the MRSA infected patients and 9.7 days (range: 1–30 days) for the MSSA infected patients.

**Table 1:** Comparison of demographic characteristics among both the groups of MRSA and MSSA infected patient

Characteristics	Categories	MRSA (n=130)	MSSA (n=130)	OR and 95% CI	p Value
Age	Above 60 years	28 (21.54%)	29 (22.31%)	0.96 (0.53, 1.72)	0.881
	Below 60 years	102 (78.5%)	101 (77.7%)		
Gender	Female	34 (26.15%)	34 (26.15%)	1 (0.58, 1.74)	1.00
	Male	96 (73.85%)	96 (73.85%)		
Emergency Admission	Present	29 (22.31%)	23 (17.69%)	1.34 (0.73, 2.46)	0.35
	Absent	101 (77.69%)	107 (77.31%)		
Immunosuppression status	Immunosuppressed	27 (20.77%)	27 (20.77%)	1 (0.55, 1.82)	1
	Non-immunosuppressed	103 (79.33%)	103 (79.33%)		
Presence of Diabetes Mellitus	Diabetic	35 (26.9%)	38 (29.2%)	1.20 (0.71, 2.02)	0.503
	Non diabetic	95 (73.1%)	92(70.8%)		
Smoking	Smoker	39 (30%)	49 (37.69%)	1.44 (0.84, 2.37)	0.19
	Non smoker	91 (70%)	81 (62.31%)		
Presence of other infections	Present	30 (23.08%)	33 (25.38%)	1.13 (0.64, 2.0)	0.664
	Absent	100 (76.92%)	97 (74.62%)		
Invasive diagnostic procedure	Performed	30 (23.08%)	30 (23.08%)	1 (0.562, 1.78)	1
	Not performed	100 (76.92%)	100 (76.92%)		
Presence of urinary catheter	Catheterized	17 (13.1%)	17 (13.1%)	1 (0.486, 2.057)	1
	Non catheterized	113 (86.9%)	113 (86.9%)		
Ryle's tube feeding	NG feed	13 (10%)	12 (9.23%)	1.09 (0.48, 2.50)	0.833
	No NG feed	117 (90%)	118 (90.77%)		
ICU admission	ICU admitted	14 (10.77%)	20 (15.38%)	1.51 (0.73, 3.13)	0.272
	No ICU admission	116 (89.33%)	110 (84.62%)		
Duration of surgery (>3 hours)	More than 3 hours	24 (18.46%)	30 (23.08%)	1.33 (0.73, 2.42)	0.359
	Less than 3 hours	106	100 (76.92%)		

Both the MRSA and MSSA infected patients were comparable (Table 1) as the odds ratio was not significant at p<0.05. Hence, the groups were considered for further statistical analysis to identify the risk factors.

The risk factors given in Table 2 were considered for multiple logistic regression since the univariate analysis indicated statistical significance. The risk factors along with the odds ratio and 95% confidence interval are given in Table 2.

**Table 2:** The risk factors through univariate logistic regression

Characteristics	Categories	MRSA (n=130)	MSSA (n=130)	OR and 95% CI	p Value
Surgical procedure performed	Surgery done	96 (73.85%)	87 (66.9%)	0.717 (4.20, 1.24)	0.021
	No surgery done	34 (26.2%)	43 (33.1%)		
Prolonged hospitalization	Prolonged hospitalization	96 (73.85%)	116 (89.23%)	2.94 (1.49,5.78)	0.001
	Early discharge	34 (26.2%)	14 (10.8%)		
Prior Antibiotic usage	History of recent antibiotic use	63 (48.46%)	44 (33.85%)	0.473 (0.285, 0.785)	0.004
	No history of recent antibiotic use	67 (41.54)	86 (66.15%)		
Presence of open wound	Had open wounds	64 (49.23%)	46 (35.38%)	1.77 (1.08, 2.91)	0.024
	No open wounds	66 (50.77%)	84 (64.66%)		
Presence of surgical drain	Surgical drain present	54 (41.54%)	34 (26.15%)	2.0 (1.19, 3.39)	0.009
	No surgical drains	76 (58.46%)	96 (73.85%)		
Presence of endotracheal tube	Presence of endotracheal tube	6 (4.62%)	1 (0.77%)	8.46 (1.04, 68.64)	0.046
	Endotracheal tube absent	124 (95.38%)	129 (99.23%)		
Presence of tracheostomy tube	Presence of tracheostomy	16 (12.31%)	6 (4.62%)	2.90 (1.10, 7.67)	0.032
	No tracheostomy	114 (87.69%)	124 (95.38 %)		
Intravenous lines	Presence of peripheral IV lines	115 (88.46%)	103 (79.23%)	0.498 (.251, .987)	0.046
	Absence of IV lines	15 (11.54%)	27 (20.77%)		
Presence of vascular/pressure ulcer	Presence of vascular or pressure ulcer	23 (17.69%)	9 (6.92%)	2.68 (1.14,6.33)	0.011
	Absence of any ulcers	107 (82.31%)	221 (93.08		
Previous recent hospitalization	Had recent hospitalization	77 (59.23%)	52 (40%)	2.18 (1.33, 3.58)	0.002
	No recent hospitalization	53 (40.77%)	78 (60%)		

Significance considered as p<0.05 level

The risk factors for MRSA infection which showed significance such as prolonged hospitalization, undergoing surgical procedures, surgical drain, previous use of antibiotics, presence of open wounds, having endotracheal and tracheostomy tubes, presence of intravenous access, presence of vascular/pressure ulcers and recent previous hospitalization were considered for further multiple logistic regression. Multiple logistic regression was adjusted with the age (advanced age) and gender (female), as these two factors are biologically significant and adjusted odds ratios are given in Table 3.

**Table 3:** Multiple logistic regression with adjusted Odds Ratio on risk factors of MRSA infection

	β coefficient	Adjusted OR	95 % CI		P
			
Risk factors			Lower	Upper	
Surgical procedure performed	1.471	4.355	1.03	18.328	0.045
Prolonged hospitalization	-1.182	0.307	0.11	0.832	0.020
Prior Antibiotic use	-0.709	0.492	0.20	1.204	0.120
Presence of open wound	0.292	1.339	0.58	3.089	0.494
Presence of surgical drain	-0.223	0.800	0.35	1.85	0.601
Presence of ET	-2.427	0.088	0.01	1.305	0.077
Presence of TT	1.667	5.298	1.16	24.298	0.032
Intravenous lines	-0.058	0.944	0.22	4.134	0.939
Pressure/venous ulcer	1.975	7.205	1.75	29.606	0.006
Recent hospitalization	1.059	2.883	1.25	6.631	0.013
Constant	-2.482				

Odds ratio adjusted to age and gender					

Logistic regression model: log (odds of MRSA) = -2.482 + 1.471 (performing surgery)+ -1.182 (prolonged hospitalization) + 1.667 (presence of tracheostomy tube) + 1.975 (presence of pressure/venous ulcer) + 1.059 (Recent hospitalization)

Out of the above risk factors, surgery as a treatment option (OR 4.355; CI 1.03, 18.328; p=0.045), prolonged hospitalization (OR 0.307; CI 0.11, 0.832; p=0.020), presence of tracheostomy tube (OR 5.298, CI 1.16, 24.298; p=0.032), presence of pressure/venous ulcer (OR 7.205; CI 1.75, 29.606; p=0.006) and previous recent hospitalization (OR 2.883; CI 1.25, 6.631; p=0.013) were significant risk factors for causing MRSA infection among hospitalized patients.

## Discussion

*Staphylococcus aureus* remains the most common pathogen causing infection in wounds^20^. World Health Organization has stressed that MRSA is one of the high priority multidrug-resistant organism^21^. MRSA infection is high in Asia and the region is considered as ‘hospital associated MRSA endemic area’^17^.

In the present study, undergoing surgery, prolonged hospitalization, presence of tracheostomy tube, pressure/venous ulcer and recent hospitalization were the significant independent risk factors causing MRSA infection among hospitalized patients.

The prevalence of MRSA infection is high among emergency admission patients^6^. In the present study, 34.9% of patients were admitted from the trauma center and have undergone emergency surgeries. Usually, emergency surgeries are not well prepared like elective surgeries. However, emergency admission was not a significant determinant in our study.

Callejo et al. and Sun et al. reported that the risk factors of MRSA infection were advanced age (above 65 years), traumatic injuries, admitted from a long-term care facility, presence of a urinary catheter, previous antibiotic treatment and skin-soft tissue or post-surgical superficial skin infections^12^,^22^. Patients with an open fracture tend to get infected more (14.7%) than a closed fracture (4.2%)^23^ or open injuries^22^. In contrast, none of these factors were significant in the present study. Therefore, it can be inferred that the risk factors of MRSA infection differ around the globe.

Patients who have undergone surgical debridement within one year (adjusted odds ratio, 2.6; 95% CI, 1.4–5.0, p=0.002) and obesity (adjusted OR 3.4, 95% CI 1.4–8.8, p=0.008) were at risk of developing recurrent MRSA infection^24^. Vascular ulcer increases the risk of MRSA infection^25^. In agreement to this, the presence of vascular ulcer in the present study was one of the significant risk of causing MRSA infection. Vascular ulcer reduces the blood flow to distal areas. In the absence of oxygen, wound healing is delayed. Non-healing of the ulcer increases the risk of infection.

In the current study, bed occupancy was more than 80%. The studies have proven that the occupancy rate in the hospital is directly proportional to the incidence of HAIs^26^. The previous hospitalization is a proven cause of MRSA bacteremia^27^. Recent hospitalization (OR 2.883; CI 1.25, 6.631; p=0.013) within a year was a significant cause of MRSA infection. Both prolonged hospitalization and repeated hospitalization increases the risk. The previous history of nursing home admission (OR 8.42; 1.06–66.43) is another threat of acquiring MRSA infection^9^. Hospital is a source of multiple pathogens, and transmission of such pathogens from the hospital to the host is common. MRSA is seen in hospital environmental surface (38.9%) which increases the risk of causing infection^17^. The ICU environment (67.3%) is an additional well-known risk factor of getting MRSA infection^7^. MRSA was detected in ventilators (33%)^7^, ultrasound transducers (17%)^28^ and stethoscopes used in the hospital^29^,^30^. Also, MRSA is detected on the hands of 59.6% healthcare professionals^7^.

Old age and nursing home residences are found to be independent risk factors of MRSA infection related death^31^. Pre-prosthetic infection with MRSA is increasing (44%) among orthopedic surgery patients^32^ and arthroplasty patients have a higher risk (OR 0.11; 0.02–0.56) than internal fixation^9^ which also increases the treatment costs. Most of the time, removal of the prosthesis is the treatment for prosthetic infection and this infection indicates the failure of treatment.

MRSA infection can have an adverse effect on the life of infected patients. The consequence of the infection can be repeated hospitalization, increased healthcare cost, increased mortality and morbidity^11^. A retrospective study carried out in Texas showed that 21% MRSA infected patients developed recurrent infection^22^. A two year retrospective study of amputated patients showed 7.3% re-hospitalization due to stump infection. Among the re-admitted patients, MRSA was the leading pathogen causing infection and the most common cause of death^33^. The occurrence of surgical site infection with MRSA among orthopedic and transplant surgery patients is in late post-operative days compared to general surgical patients^34^. This indicates that a longer duration of hospitalization is a threat for the development of infection. Longer hospitalization not only causes wound infection but also can result in MRSA bacteremia. In the present study, prolonged hospitalization (OR 0.307; CI 0.11, 0.832; p=0.020) was a significant contributing factor of MRSA infecton. The mean duration of the hospitalized MRSA infected patients was 9.9 days. The duration of the hospitalization differs for each disease condition. However, for patients with minor surgeries, more than three days of hospitalization and more than seven days for major surgeries were considered as prolonged hospitalization. For patients, without surgical procedures (only medical treatment) the duration of hospitalization was compared with our hospital policy.

In the present study, undergoing surgery emerged as a risk factor. As surgical procedure disrupts the integrity of the skin, a pathogen can enter into the body easily. It is also noted that, more personnel in the operation room increases the risk of infection^35^. However, the operating room team is bigger in teaching hospitals as students are posted in the operation room to develop surgical skills. Therefore, additional measures need to be implemented to reduce risk.

Presence of endotracheal or tracheostomy tubes and vascular ulcers result in infection. Though ulcers can be prevented, managing the patient with endotracheal or tracheostomy is unavoidable in many situations. Therefore, additional emphasis is needed for infection control. These patients need to stay for a longer time in the hospital. A systematic review revealed that the cost of treating MRSA infection is high^36^. Though hospitalization cannot be completely eliminated, the hospital must take necessary measures to reduce the duration of hospitalization and avoid repeated admissions.

## Limitation

The study conducted at a single center with convenient sampling lacks the generalizability. Perhaps further studies are required covering diverse geographical and clinical areas which may help in developing appropriate guidelines to prevent MRSA infection.

## Conclusion

We identified that the damage to the skin and mucosal barriers such as undergoing surgical procedures and the existence of pressure or venous ulcers increase the risk of acquiring MRSA infection. Prolonged length of hospital stay and the history of recent hospitalization are the other risk factors. In addition, tracheostomy escalates the threat of MRSA infection in wounds of patients admitted to the hospital. Hence, controlling these risk factors may help in reducing the burden of infection.

## References

[1] Raza M, Kazi B, Mustafa M, Gould F (2014). Developing countries have their own characteristic problems with infection control. J Hosp Infect.

[2] Guleri A, Kehoe A, Hartley J, Lunt B, Harper N, Palmer R (2011). The costs and benefits of hospital MRSA screening. British Journal of Health Care Management.

[3] Kao KC, Chen CB, Hu HC, Chang HC, Huang CC, Huang YC (2015). Risk factors of methicillin-resistant Staphylococcus aureus infection and correlation with nasal colonization based on molecular genotyping in medical intensive care units: a prospective observational study. Medicine.

[4] Center for Disease Control and Prevention (2019). Methicillin-resistant Staphylococcus aureus (MRSA).

[5] Dadashi M, Nasiri M, Fallah F, Owlia P, Hajikhani B, Emaneini M (2018). Methicillin-resistant Staphylococcus aureus (MRSA) in Iran: A systematic review and meta-analysis. J Glob Antimicrob Resist.

[6] Davis C, Stoppler MC (2019). Methicillin-Resistant Staphylococcus Aureus (MRSA).

[7] Tajeddin E, Rashidan M, Razaghi M, Javadi S, Sherafat S, Alebouyeha M (2016). The role of the intensive care unitenvironment and health-care workers in the transmission of bacteria associated with hospital acquired infections. J Infect Public Health.

[8] Al-Mulhim FA, Baragbah MA, Ali MS, Abdallah SA (2014). Prevalence of Surgical Site Infection in Orthopedic Surgery: A 5-year Analysis. Int Surg.

[9] Deny A, Loiez C, Deken V, Putman S, Duhamel A, Girard J (2016). Epidemiology of patients with MSSA versus MRSA infections of orthopedic implants: Retrospective study of 115 patients. Orthop Traumatol Surg Res.

[10] Boswihi S, Udo E (2018). Methicillin-resistant Staphylococcus aureus: An update on the epidemiology, treatment options and infection control. CMRP.

[11] Calfee D (2017). Trends in Community Versus Health Care-Acquired Methicillin-Resistant Staphylococcus aureus Infections. Curr Infect Dis Rep.

[12] Callejo TF, Eiros BJM, Olaechea AP, Coma Del MJ, Palomar MM, Alvarez LF (2016). Risk factors for methicillin-resistant Staphylococcus aureus colonisation or infection in intensive care units and their reliability for predicting MRSA on ICU admission. Infez Med.

[13] Mundhada SA, Tenpe S (2015). A study of organisms causing surgical site infections and their antimicrobial susceptibility in a tertiary care Government Hospital. Indian J Pathol Microbiol.

[14] Bearman G, Bryant K, Leekha S, Mayer J, Munoz-Price S, Murthy R (2014). Healthcare personnel attire in non-operating-room settings. Infect. Control Hosp. Epidemiol.

[15] Hefzya E, Wegdana A, Wahed W (2016). Hospital outpatient clinics as a potential hazard for healthcare associated infections. J Infect Public Health.

[16] Bogusz A, Stewart M, Hunter J, Yip B, Reid D, Robertson C (2013). How quickly do hospital surfaces become contaminated after detergent cleaning?. Healthc Infect.

[17] Chen CJ, Huang YC (2014). New epidemiology of Staphylococcus aureus infection in Asia. Clin Microbiol Infect.

[18] Graffunder EM, Venezia RA (2002). Risk factors associated with nosocomial methicillin-resistant Staphylococcus aureus (MRSA) infection including previous use of antimicrobials. J Antimicrob Chemother.

[19] Wayne P (2015). Performance Standards for Antimicrobial Susceptibility Testing: Twenty-Fifth Informational Supplement.

[19] Graffunder EM, Venezia RA (2002). Risk factors associated with nosocomial methicillin-resistant Staphylococcus aureus (MRSA) infection including previous use of antimicrobials. J Antimicrob Chemother.

[20] Oladeinde BH, Omoregie R, Olley M, Anunibe JA, Onifade AA (2013). A 5-year surveillance of wound infections at a rural tertiary hospital in Nigeria. Afr Health Sci.

[21] World Health Organisation (2017). WHO publishes list of bacteria for which new antibiotics are urgently needed.

[22] Sun Y, Wang H, Tan Y, Zhao H, Qin S, Lihui XL (2018). Incidence and risk factors for surgical site infection after open reduction and internal fixation of ankle fracture. Medicine.

[23] Zalavras C (2017). Prevention of Infection in Open Fractures. Infect Dis Clin North Am.

[24] Sreeramoju P, Porbandarwalla NS, Arango J, Latham K, Dent DL, Stewart RM (2011). Recurrent skin and soft tissue infections due to methicillin-resistant Staphylococcus aureus requiring operative debridement. Am J Surg.

[25] Wang SH, Sun ZL, Guo YJ, Yang BQ, Yuan Y, Wei Q (2010). Meticillin-resistant Staphylococcus aureus isolated from foot ulcers in diabetic patients in a Chinese care hospital: risk factors for infection and prevalence. J Med Microbiol.

[26] Kaier K1, Mutters NT, Frank U (2012). Bed occupancy rates and hospital-acquired infections--should beds be kept empty?. Clin Microbiol Infect.

[27] Shuping L, Kuonza L, Musekiwa A, Iyaloo S (2017). Hospital-associated methicillin-resistant Staphylococcus aureus: A cross-sectional analysis of risk factors in South African tertiary public hospitals. PloS One.

[28] Lawrence M, Blanks J, Ayala R, Talk D, Macian D, Glasser J (2014). Hospital-wide survey of bacterial contamination of point-of-care ultrasound probes and coupling gel. J Ultrasound Med.

[29] Campos MA, Leon LX, Munoz J, Macías A, Alvarez J (2014). Stethoscopes as potential intra hospital carriers of pathogenic microorganisms. Am J Infect Control.

[30] Uneke C, Ndukwe C, Nwakpu K, Nnabu R, Ugwuoru C, Prasopa PN (2014). Stethoscope disinfection campaign in a Nigerian teaching hospital:results of a before-and-after study. J Infect De C'tries.

[31] Pastagia M, Kleinman LC, de la Cruz EG, Jenkins SG (2012). Predicting risk for death from MRSA bacteremia. Emerg Infect Dis.

[32] Siddiqui MM, Lo NN, Rahman SA, Chin PL, Chia SL, Yeo SJ (2013). Two-year outcome of early deep MRSA infections after primary total knee arthroplasty: a joint registry review. Two-year outcome of early deep MRSA infections after primary total knee arthroplasty: a joint registry review. J Arthroplasty.

[33] de Godoy JM, Ribeiro JV, Caracanhas LA, de Fatima GG (2010). Hospital infection after major amputations. Ann Clin Microbiol Antimicrob.

[34] Korol E, Johnston K, Waser N, Sifakis F, Jafri HS, Lo M (2013). A systematic review of risk factors associated with surgical site infections among surgical patients. PloS One.

[35] Maksimovic J, Denic LM, Bumbasirevic M, Vlajinac H, Marinkovic J (2008). Surgical Site Infections in Orthopedic Patients: Prospective Cohort Study. Croat Med J.

[36] Farbman L, Avni T, Rubinovitch BL, Paul M (2013). Cost-benefit of infection control interventions targeting methicillin-resistant Staphylococcus aureus in hospitals: Systematic review. Clin Microbiol Infect.

